# Qualitative and Quantitative Health‐Related Quality‐of‐Life Outcomes From an 8‐year Follow‐Up After Gastric Bypass Surgery in People With Type 2 Diabetes

**DOI:** 10.1002/edm2.70315

**Published:** 2026-08-19

**Authors:** Giovanni Fanni, Petros Katsogiannos, Magnus Sundbom, Janeth Leksell, Eva Randell

**Affiliations:** ^1^ Department of Medical Sciences, Clinical Diabetology and Metabolism Uppsala University Uppsala Sweden; ^2^ Department of Surgical Sciences Uppsala University Uppsala Sweden; ^3^ Department of Social Work Uppsala University Uppsala Sweden

**Keywords:** health‐related quality of life, obesity surgery, Type 2 diabetes

## Abstract

**Background:**

Obesity and Type 2 diabetes (T2D) impair health‐related quality of life (HRQoL) across metabolic, mechanical and mental domains. Gastric bypass surgery improves HRQoL in the short term, but long‐term trajectories remain insufficiently understood. This study evaluated HRQoL 8 years after gastric bypass in a cohort with T2D, integrating quantitative assessments with qualitative exploration of lived experiences.

**Methods:**

Eleven individuals with obesity and T2D who underwent gastric bypass participated in an 8‐year follow‐up. Anthropometric and clinical data were collected. HRQoL was assessed using the RAND SF‐36, and changes over time were analyzed using repeated‐measures ANOVA. Correlations between changes in BMI, HbA1c and HRQoL domains were assessed with Spearman correlations. Semi‐structured qualitative interviews were conducted, transcribed verbatim and analyzed using reflexive thematic analysis.

**Results:**

Participants experienced modest weight regain but significant deterioration in glucose control from the 2‐year to the 8‐year follow‐up. RAND SF‐36 scores returned to preoperative levels, and the mental health score was significantly reduced compared to the 2‐year follow‐up (*q* < 0.01). Weight regain correlated with reduced physical functioning, role‐physical and vitality scores (all *r* < −0.7, *p* < 0.05), while HbA1c increase correlated with poorer mental health and role‐emotional scores (all *r* < −0.6, *p* < 0.05). Qualitative interviews revealed that long‐term HRQoL was dynamic and shaped by an interplay of physical functioning, psychological wellbeing, body adaptations, social support and life events. An overarching theme emerged: *Quality of life after gastric bypass is a multidimensional, evolving process influenced by changing health, life circumstances and psychosocial factors over time*.

**Conclusions:**

Initial postoperative improvements in HRQoL were not sustained at 8 years and might have been influenced by weight regain, T2D relapse and intercurrent illness. However, lived experiences highlighted meaningful and durable benefits in physical functioning and self‐perception for several participants. The findings underscore the need for long‐term, holistic follow‐up that integrates metabolic monitoring with psychological and social support.

**Trial Registration:**

ClinicalTrials.gov registration: NCT02729246

## Graphical abstract

Quality of life after gastric bypass is a multidimensional, evolving process influenced by changing health, life circumstances and psychosocial factors over time, including: physical functioning, psychological well‐being, body adaptation, social supports and life events.

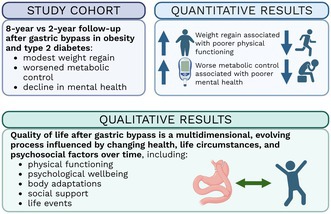

## 1 Introduction

Obesity is a chronic disease that has reached pandemic proportions [1] and is associated with substantial morbidity across metabolic, mechanical and mental health domains.

Among adults with Type 2 diabetes mellitus (T2D), cardiovascular disease (CVD) remains the leading cause of death [2]. For individuals with T2D and severe obesity, achieving therapeutic goals is often challenging, also because mobility limitations and musculoskeletal pain make it difficult to adhere to behavioural interventions, such as increasing physical activity [3]. Although individualized guidance on diet and physical activity is a cornerstone of diabetes care, long‐term weight maintenance remains difficult. The Look AHEAD trial showed how even an intensive lifestyle programme can lead to clinically significant weight loss but is then shadowed by weight regain and worse cardiovascular risk in the long‐term follow‐up, demonstrating the challenges of sustaining results over time [4].

Given these challenges, there has been growing interest in pharmacological options that could help address both obesity and diabetes simultaneously. Incretin‐based therapies have emerged as important treatment options for both obesity and T2D, although long‐term durability, comparative effectiveness and safety continue to be evaluated [5]. However, pharmacotherapy is only one part of the therapeutic landscape, and surgical interventions continue to play a major role.

Obesity surgery leads to considerable weight loss and sustained weight maintenance, alleviating the burden of obesity‐related diseases and complications. It also leads to outstanding improvements in cardiometabolic health, being able to prevent the onset or even induce the remission of T2D [6]. Building on this evidence, recent long‐term studies have begun to clarify how such surgical treatments affect the patient's lived experience and health‐related quality of life (HRQoL) over time. Recently, a large long‐term randomized study reported HRQoL outcomes after obesity surgery in individuals with obesity and T2D and found better outcomes than medical therapy in several domains [7].

We have carried out a study on a smaller cohort of people with obesity and T2D who underwent gastric bypass surgery. The study participants were extensively metabolically characterized, with a follow‐up up to 9 years after surgery (unpublished data, under review). We have previously published the HRQoL outcomes of this cohort up to 2 years after the intervention [8]. In this paper, we present a long‐term follow‐up of health‐related quality of life in this cohort, approximately 8 years after gastric bypass, complementing quantitative assessments with semi‐structured qualitative interviews to gain deeper insight into the participants' experiences.

## 2 Methods

This study presents a follow‐up analysis regarding HRQoL outcomes in a cohort of people with obesity and T2D approximately 8 years after undergoing gastric bypass surgery. Details about the study can be found in previous publications [8, 9, 10, 11].

Eleven participants took part in this substudy. However, anthropometric and clinical data were available only from 9 participants and were retrieved from the participants' case report forms. There were no missing data points in the datasets. The participants were asked to fill out the HRQoL questionnaire RAND SF‐36, in line with what was done with previous follow‐ups [8]. Also, one‐to‐one qualitative interviews were performed.

### 2.1 RAND SF‐36

The RAND SF‐36 is a widely used questionnaire to perform quantitative assessment of general HRQoL, and it consists of 36 items that evaluate eight health domains: physical functioning, role limitations due to physical health, role limitations due to emotional problems, social functioning, emotional well‐being, energy/fatigue (vitality), pain and general health perceptions. The questionnaire captures both subjective well‐being and functional limitations across physical, mental and social dimensions of health. Scores for each domain are given on a 0–100 scale, with higher scores indicating better perceived health status [12].

### 2.2 Qualitative Interviews

Individual interviews were conducted at the hospital (*n* = 2) or by telephone (*n* = 9) by the interviewer (ER). The interviews followed a study‐specific, semi‐structured interview guide (which can be found in the Table S1), complemented by probing questions (‘Can you tell me more about… or can you give an example of…’) to explore the participants' descriptions further and to ask for concrete examples. The interviews lasted between 20 and 60 min. The interviews were conducted once with each participant, recorded and transcribed verbatim.

### 2.3 Analysis of Quantitative Data and Statistical Analyses

Continuous data are presented as median (interquartile range). Analyses of variance were performed to assess longitudinal changes in HRQoL domains with multiple repeated‐measure one‐way ANOVAs. Sphericity was not assumed, and the Geisser–Greenhouse correction was applied. The presence of strong outliers was excluded via inspection of the datasets. The normal distribution of the dependent variable was confirmed via visual inspection of the Q‐Q plots. Pairwise comparisons in the ANOVA models were performed by calculating the false discovery rate according to the Benjamini, Krieger and Yekutieli method. Adjusted q‐values below 0.05 were considered statistically significant. Correlation analyses between changes in BMI, HbA1c and HRQoL domains were assessed using Spearman correlations.

### 2.4 Analysis of Qualitative Data

All transcribed data were analyzed using thematic analysis, a method for identifying, analyzing and reporting recurring patterns (themes) [13]. The interviews were analyzed by two of the current authors (ER, JL), who began by reading the transcripts separately to gain an overview of the content. Sentences related to quality of life were marked. In the second phase, text segments were marked with descriptive data‐driven codes. In the third phase, once all interviews had been coded, the codes were extracted, reviewed jointly and grouped into subthemes. In the final step of the analysis, the overarching theme and sub‐themes were reviewed and discussed among the authors. The analysis was performed manually in a word processing program; no special analysis software was used.

### 2.5 Ethics Approval

The study was approved by the Research Ethics Committee at Uppsala University (DNR 2014/255, DNR 2023‐08148‐02) and complied with the Declaration of Helsinki. All participants gave their written informed consent after they had been informed about the study's purpose, that participating in the study was voluntary and that they could terminate participation without any consequences.

## 3 Results

Of the 13 original participants who underwent gastric bypass, 11 agreed to participate in the qualitative interview, whilst 9 agreed to participate in the clinical follow‐up and filled in the RAND SF‐36 questionnaire. Time since surgery ranged from 7 to 9 years (mean 8.0 years). Seven participants had a relapse of T2D. In general, participants regained weight since the 2‐year follow‐up (BMI from 29.2 [26.6–31.5] to 32.3 [26.7–33.9], *p* = 0.042) and their glycaemic control deteriorated (HbA1c from 38 [37–46] to 46 [40–58], *p* = 0.046), although median BMI and HbA1c remained below baseline values. More details about the clinical characteristics of the cohort can be found elsewhere (unpublished data, under review).

### 3.1 RAND SF‐36

**FIGURE 1:**
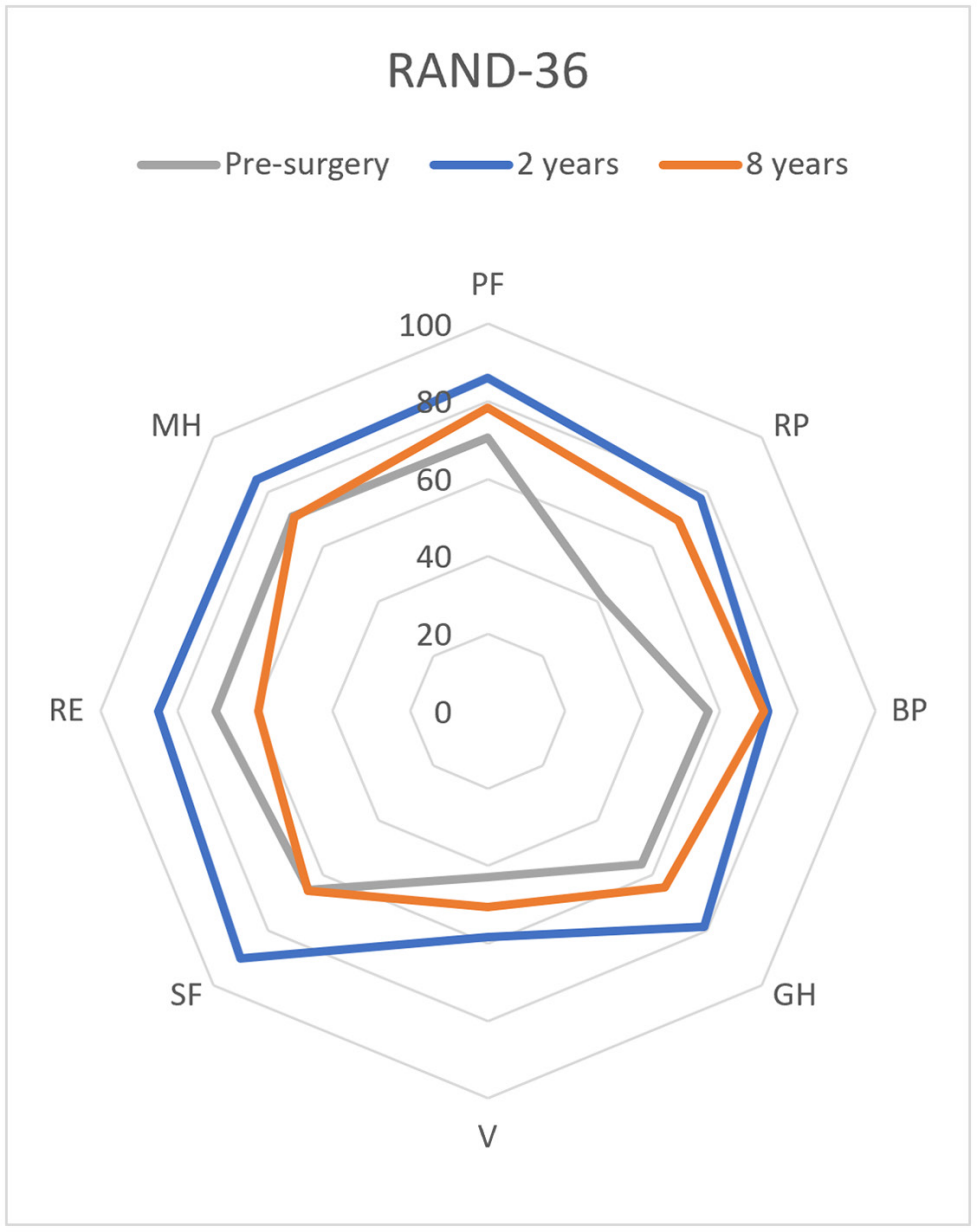
Results from the RAND‐36 scores across all domains at baseline, 2 years and 8 years after surgery. Data are presented as means. BP, Bodily pain; GH, General health; MH, Mental health; PF, Physical functioning; RE, Role‐functioning/emotional; RP, Role‐functioning/physical; SF, Social functioning; V, Vitality (energy and fatigue).

Compared to the 2‐year follow‐up, the participants showed a significant decline in the domain of mental health (*q* < 0.01). Overall, the scores in all domains were not statistically different from those obtained before the surgical intervention. Specific scores for all RAND domains are reported in Figure 1.

It needs to be remarked that some of the study participants suffered from severe clinical conditions since the previous follow‐up, including cardiovascular events and chronic complications related to diabetes, which have likely affected their overall perception of HRQoL.

A higher increase in HbA1c from 2 to 8 years after surgery correlated with a lowering in scores for mental health (*r* = −0.761, *p* = 0.02) and role‐emotional (*r* = −0.696, *p* = 0.04). The increase in BMI correlated instead with worse scores in physical functioning (*r* = −0.762, *p* = 0.02), role‐physical (*r* = −0.904, *p* = 0.002) and vitality (*r* = −0.865, *p* = 0.005).

### 3.2 Qualitative Interviews

An overarching theme and five sub‐themes were identified from interviews.

Overarching theme: Quality of life after obesity surgery is a dynamic process shaped by changing health, life events and social and psychological factors over time.

Subthemes: (1) Physical functioning; (2) Body adaptations and everyday strategies; (3) Psychological functioning; (4) Social support; (5) In the shadow of life changes.

From the participants' descriptions, quality of life emerged as a multidimensional phenomenon that encompassed several factors. Overall, the analysis suggested that quality of life after obesity surgery was often positive but not static. It was continuously shaped by new life events such as illness, ageing or changes in lifestyle habits, and several participants experienced a recurrence of diabetes in the long term. Quality of life emerged from the interaction between physical functioning, psychological functioning, strategies to deal with everyday life, social support, life circumstances and current health status. It was therefore best understood from a life‐course perspective, where ageing, new illnesses and changing life conditions interacted with the long‐term effects of surgery. The main findings for each subtheme are summarized below.

#### 3.2.1 Physical Functioning

Participants frequently described a profound sense of life satisfaction after surgery, where improved vitality and physical functioning became central to their renewed quality of life. Many portrayed the decision to undergo surgery as both autonomous and carefully considered, and the subsequent weight loss emerged as a stable, long‐term resource that strengthened their everyday functioning and work capacity. A wide range of positive changes followed. Participants spoke of newfound energy, deeper and more restorative sleep, and work situations that felt more manageable and fulfilling. The health benefits were equally striking: some were able to discontinue diabetes medication entirely, while others significantly reduced their treatment. The substantial weight loss also opened the door to increased physical activity and more sustainable lifestyle habits, which several participants described as both empowering and transformative.

For one participant, a relatively large and stable weight loss enabled long‐term mobility:

I go to the gym three times a week. I have chores at home, as I am retired. You live in a villa, so there is a lot to do with the house. I have built a garage that I am finishing. I am renovating my motorcycle. I try to live an active life. (Participant 1)

Regarding perceived physical strength, one participant stated that the surgery had contributed to increased physical capacity and stable weight over time:

I am in the best shape of my life. (Participant 9)

#### 3.2.2 Body Adaptations and Everyday Strategies

After surgery, participants encountered new physical challenges such as blood sugar dips and dumping, which required increased body awareness and daily adjustments. They learned to interpret bodily signals and manage symptoms proactively. Quality of life was described not as the absence of problems, but as the ability to handle them. One participant noted how unclear hunger signals demanded constant meal planning to prevent weakness and shakiness.

To maintain weight control, several participants used goal‐oriented strategies, such as regular self‐monitoring:

I still weigh myself a couple of times a week. If it's going up, then I try to do something. I deal with it right away. (Participant 6)

Dietary adaptations included portion control, smaller plates and identifying personal tolerances. Internal motivation was central:

Now I eat a small bowl of chips. That's enough because then I'm satisfied. […] I haven't become a fanatic, but it has worked well. The most important thing is to find a way you want to live. (Participant 9)

Participants emphasized balancing discipline with flexibility, allowing occasional treats without losing control. One described managing cravings by limiting intake or simply observing foods without buying them:

I look at chocolate with my eyes but don't buy it. At least I've seen it. (Participant 3)

Not all strategies were beneficial. One participant developed increased alcohol use after surgery but later addressed it with medical support. For those with limited physical capacity, practical adaptations supported independence. As one participant explained:

I try to be outside every day but there are no long walks. […] I order everything we need online. (Participant 4)

#### 3.2.3 Psychological Functioning

The quality of life was also shaped by psychological processes such as managing expectations and renegotiating identity and wellbeing over time. Before surgery, life was often characterized by the experience of social scrutiny related to overweight. In the years following surgery, weight loss reduced internalized stigma and increased body acceptance. However, some participants described a remaining ‘internal overweight identity’, suggesting a long‐term ongoing transitional process. Quality of life was therefore associated with improved self‐esteem and greater body acceptance.

I felt better and friends thought it was great that I was doing well. In the beginning, […] many people didn't even recognize me, and I had to explain who I was. (Participant 6)

Psychological functioning could also be influenced by fear of illness. One participant had lived without diabetes for 4 years after surgery, but when the disease relapsed it triggered renewed anxiety despite the sustained weight loss:

After the surgery my mental health was back to normal. After I found out I had diabetes, I stopped eating sugar and cut out everything with sugar. […] Sometimes my kids say, ‘take a bite,’ but I say no. I've lost my sister and my mother to diabetes, so I don't dare. (Participant 3)

Several participants highlighted trust in healthcare and the security of continued follow‐up through research participation as important for emotional wellbeing. This trust contributed to a sense of safety and reassurance.

#### 3.2.4 Social Support

Social quality of life was closely tied to family support, especially from partners. Stable, long‐term relationships emerged as a key foundation for wellbeing, where health improvements were strengthened through social affirmation, shared routines and mutual influence within the family.

Support from partners and children played a central role, and for those still working, encouragement from colleagues and friends further reinforced a sense of social wellbeing. Positive comments, recognition of weight loss, and everyday affirmations contributed to a strengthened social identity and increased confidence.

Partner support in everyday life was particularly emphasized. One participant described how her husband supported her both before and after surgery:

We who are with each other daily, 24‐7. The most important thing is that he supports me. And he did. He supported me before the surgery—he even ate that disgusting Modifast so I wouldn't have to cook separately. And later he has continued to support me wholeheartedly. (Participant 9)

#### 3.2.5 In the Shadow of Life Changes

Most participants experienced age‐related health problems such as arthritis, back pain, or rheumatism. Some had also experienced more serious illnesses that significantly affected their quality of life. One participant described how daily life and wellbeing were influenced by the interaction between physical limitations, chronic illness, social responsibilities and personal coping strategies. Despite earlier improvements in diabetes and weight loss, current physical health was significantly impaired:

It has nothing to do with the surgery but with long‐term COVID. I get a fever when I do the slightest thing. My diabetes is better, but it's hard to distinguish what is due to long‐term COVID and what is due to the surgery. I am actually sicker today than I was before the surgery. (Participant 4)

Another participant, who had experienced cancer, distinguished between the effects of surgery and other illnesses:

When it comes to the weight loss and surgery, I feel really good. Then I have other problems with cancer that I have follow‐ups for. I've had surgery twice for malignant melanoma. But the surgery itself went very well, both with food and exercise. (Participant 1)

These accounts illustrate that quality of life was situational and strongly influenced by current health status rather than solely by long‐term surgical outcomes.

## 4 Discussion

Altogether, our results indicate that initial improvements in HRQoL following gastric bypass were not sustained at the 8‐year follow‐up. Overall, HRQoL returned to levels similar to those observed preoperatively, with a notable deterioration in the mental health domain. These findings align with previous systematic reviews and longitudinal studies indicating that initial improvements in HRQoL after obesity surgery may diminish over time, particularly in the context of weight regain and metabolic relapse [14, 15]. In our cohort, most participants experienced relapse of T2D despite moderate weight regain. These changes have contributed to deterioration in selected HRQoL domains, underscoring the importance of long‐term follow‐up [15].

In a large study including participants with obesity and T2D randomized to either obesity surgery or medical and lifestyle intervention, HRQoL assessed with RAND‐36 showed that obesity surgery led to a peak improvement within 3 years after the intervention in the physical component score, followed by a progressive decrease returning towards baseline levels at the 12‐year follow‐up. On the other hand, scores in mental health, social functioning and role‐emotional showed a decreasing trend throughout the follow‐up [7]. Our results are in line with published literature, showing peak improvements in HRQoL 2 years after surgery and a return to baseline levels 8 years after the intervention.

Remarkably, weight regain was associated with worsened perceived physical functioning and vitality, while worsened diabetes control was associated with worse emotional functioning. To the best of our knowledge, we are the first to report these correlations, although no causal associations can be inferred due to the observational nature of the study as well as the small sample size.

At the same time, qualitative findings highlight that HRQoL after obesity surgery cannot be fully captured by quantitative measures alone. Participants' narratives illustrate that quality of life is dynamic and life‐course dependent, shaped by the interplay of physical health, psychological factors, social relationships and life events [16]. Thematic analysis suggests that post‐surgical quality of life is a dynamic process shaped by changing health, life events and social and psychological factors over time. These data complement the quantitative findings and help explain fluctuations in HRQoL despite persistent physiological effects of surgery.

Despite objective deterioration in HbA1c and partial weight regain, several participants reported enduring improvements in physical function, energy and work capacity. For some, weight loss enabled a more active lifestyle which was maintained over the years. These observations echo previous qualitative studies showing that physical function and perceived well‐being are influenced not only by weight or metabolic parameters but also by motivation, activity and self‐perceived bodily capacity [17, 18]. Recognizing this is clinically important, as traditional assessments often prioritize objective metrics over subjective experiences.

Psychological factors were central to long‐term quality of life. Many participants described reduced internalized stigma, enhanced self‐esteem and greater bodily acceptance, while others retained aspects of an ‘internal obesity identity’, suggesting that identity transformation following obesity surgery is gradual and complex [19]. T2D relapse elicited worries and negatively impacted psychological well‐being, particularly for those with a familial history of diabetes‐related complications. These findings reinforce previous work indicating that body image and obesity identity play crucial roles in post‐surgical outcomes [19].

Participants also developed strategies to manage bodily changes after surgery, including coping with dumping, regulating hunger and adjusting portion sizes, fostering a sense of control. On the other side, one participant developed an alcohol use disorder, a known possible complication of gastric bypass [20] and with the highest prevalence in younger women [21, 22]. It is therefore of high clinical importance to monitor behavioural adaptations after obesity surgery, in addition to weight and metabolic outcomes [23].

Social support emerged as a critical factor for both physical and psychological quality of life. Support from partners, family and friends was reported to enhance motivation, facilitate lifestyle adjustments and provide emotional stability, consistent with previous research emphasizing the centrality of social relationships in long‐term post‐obesity well‐being [24].

Comorbidities and life events also played a pivotal role in shaping quality of life. Several participants experienced serious illnesses, such as cancer, long COVID, or sepsis, which influenced daily functioning and health perceptions independently of surgical outcomes. This highlights the complexity of disentangling the long‐term effects of obesity surgery from the broader context of an individual's life, especially in the long‐term follow‐up.

This study is limited by a small sample size and potential selection bias, which can affect the generalizability of the results. On the other side, the study is strengthened by the integration of quantitative and qualitative approaches, which allowed for a more nuanced understanding of the evolution of HRQoL over time. While the RAND SF‐36 captures key HRQoL domains, it does not encompass all dimensions relevant to patients who had obesity surgery, as demonstrated by the qualitative data.

## 5 Conclusions

We showed that in individuals with obesity and T2D undergoing gastric bypass surgery, the early improvements in HRQoL were not maintained at 8 years, with quantitative HRQoL scores returning to preoperative levels and mental health declining significantly. Weight regain and relapse of diabetes were linked to poorer HRQoL outcomes. Nevertheless, qualitative accounts revealed that many participants continued to experience meaningful benefits in physical functioning, daily life and self‐perception. Taken together, our findings highlight that long‐term HRQoL after gastric bypass is shaped not only by metabolic outcomes but also by life events, psychosocial factors and evolving coping strategies. Sustained, multidisciplinary postoperative support is essential to address the complex and dynamic needs of this patient population.

## Author Contributions

**Petros Katsogiannos:** conceptualization, investigation, writing – review and editing. **Magnus Sundbom:** resources, writing – review and editing. **Janeth Leksell:** conceptualization, investigation, writing – review and editing, writing – original draft, methodology. **Giovanni Fanni:** conceptualization, investigation, writing – original draft, writing – review and editing, visualization, formal analysis, data curation. **Eva Randell:** writing – review and editing, writing – original draft, methodology, formal analysis, conceptualization, investigation.

## Funding

This work was funded by the Swedish Diabetes Foundation (DIA2021‐661, DIA2024‐935), the Swedish National Strategic Research Initiative EXODIAB (Excellence of Diabetes Research in Sweden), the Family Ernfors Foundation, the P.O. Zetterlings Foundation, the NovoNordisk Foundation (NNF23OC0084483, NNF25OC0101843), the Agnes and Mac Rudberg Foundation, the European Commission Horizon RIA project PAS GRAS (101080329), the Swedish Foundation for Strategic Research, the Swedish Research Council (2024‐03344), Uppsala University Hospital ALF grants.

## Ethics Statement

Ethical approval was obtained by the Regional Ethics Review Authority in Uppsala, Sweden, with registration number DNR 2014/255 and following amendment (DNR 2023‐08148‐02). The study has been conducted in accordance with the principles of the Declaration of Helsinki. Participants have received written and oral information about the study procedure and signed an informed consent form before starting any study investigation.

## Consent

The authors have nothing to report.

## Conflicts of Interest

The authors declare no conflicts of interest.

## Supporting information


**Table S1:** Interview guide used at the 8‐year follow‐up after gastric bypass.

## Data Availability

The data that support the findings of this study are available from the corresponding author upon reasonable request.
