# Supplementary material for: Qualitative and Quantitative Health‐Related Quality‐of‐Life Outcomes From an 8‐year Follow‐Up After Gastric Bypass Surgery in People With Type 2 Diabetes
Source: Endocrinol Diabetes Metab. 2026 Aug 19;9(5):e70315. doi: 10.1002/edm2.70315 (PMC13487693; doi:10.1002/edm2.70315)
Supplement: Supplementary file 1 [file EDM2-9-e70315-s001.docx]

**Supplementary material**

**Table S1**. Interview guide used at the 8-year follow-up after gastric bypass.

1. Can you tell me how you are feeling today?

2. Can you tell me about how you live your life today?

3. How is your physical health today?

4. How is your mental health today?

5. Is anything better in your life today compared to before the operation?

6. Is anything worse in your life today compared to before the operation?

7. Do you think the improvements/deteriorations can be linked to the operation itself, or is it due to something else?

8. How has your life been in general in the years since the operation?

9. What challenges have you faced in your life since the operation?

10. How have you dealt with these challenges?

11. How has your physical health been in the years since the operation?

12. How has your mental health been in the years since the operation?

13. Has your life changed as a result of the operation? If so, in what way?

14. How has your social situation (family, work, employment) been since the operation?

15. Has your view of yourself been affected? If so, in what way?

16. Has other people's view of you been affected? If so, in what way?

17. How do you remember the time before the operation now?

18. How do you feel about the support you have received after the operation (from family, friends, work, healthcare, etc.)?

19. Has the support changed over time in the years since the operation? If so, how? (E.g., more at the beginning? The same over time? Difference between specialist care/primary care?)

20. What is important to you when it comes to lifestyle habits?

21. Based on what you know today, what would you have liked to know before the operation?

22. What would you like to change to improve care and support?

23. Is there anything else you would like to share or add before we conclude?
